# [Retracted] p73 participates in WWOX-mediated apoptosis in leukemia cells

**DOI:** 10.3892/ijmm.2026.5925

**Published:** 2026-07-13

**Authors:** Donghong Lin, Zhaolei Cui, Lingying Kong, Feng Cheng, Jianping Xu, Fenghua Lan

Int J Mol Med 31: 849-854, 2013; DOI: 10.3892/ijmm.2013.1289

Following the publication of the above article, a concerned reader drew the Editor's attention to the fact that, regarding the the western blot data shown in Fig. 4C on p. 853 for the Jurkat cell line, the p73 bands shown to portray the 72 and 96 h experiments for pGC-FU-WWOX were strikingly similar to the p73 bands shown to represent the 48 and 72 h experiments for pGC-FU-GFP. Upon assessing these data in the Editorial Office, it was concluded that the concerns of the reader were legitimate, suggesting that this figure may have been inappropriately edited.

The Editor of *International Journal of Molecular Medicine* has therefore decided that the article should be retracted from the Journal on the grounds of an overall lack of confidence in the data. The authors were asked for an explanation to account for these concerns, but the Editorial Office did not receive a satisfactory reply. The Editor sincerely apologizes to the readership for any incovenience caused, and we thank the reader for drawing this matter to our attention.

